# Differential control of dNTP biosynthesis and genome integrity maintenance by the dUTPase superfamily enzymes

**DOI:** 10.1038/s41598-017-06206-y

**Published:** 2017-07-20

**Authors:** Rita Hirmondo, Anna Lopata, Eva Viola Suranyi, Beata G. Vertessy, Judit Toth

**Affiliations:** 1 Institute of Enzymology, RCNS, Hungarian Academy of Sciences, Budapest, Hungary; 2 Department of Applied Biotechnology, Budapest University of Technology and Economics, Budapest, Hungary

## Abstract

dUTPase superfamily enzymes generate dUMP, the obligate precursor for *de novo* dTTP biosynthesis, from either dUTP (monofunctional dUTPase, Dut) or dCTP (bifunctional dCTP deaminase/dUTPase, Dcd:dut). In addition, the elimination of dUTP by these enzymes prevents harmful uracil incorporation into DNA. These two beneficial outcomes have been thought to be related. Here we determined the relationship between dTTP biosynthesis (dTTP/dCTP balance) and the prevention of DNA uracilation in a mycobacterial model that encodes both the Dut and Dcd:dut enzymes, and has no other ways to produce dUMP. We show that, in *dut* mutant mycobacteria, the dTTP/dCTP balance remained unchanged, but the uracil content of DNA increased in parallel with the *in vitro* activity-loss of Dut accompanied with a considerable increase in the mutation rate. Conversely, *dcd:dut* inactivation resulted in perturbed dTTP/dCTP balance and two-fold increased mutation rate, but did not increase the uracil content of DNA. Thus, unexpectedly, the regulation of dNTP balance and the prevention of DNA uracilation are decoupled and separately brought about by the Dcd:dut and Dut enzymes, respectively. Available evidence suggests that the discovered functional separation is conserved in humans and other organisms.

## Introduction

**Figure 1:**
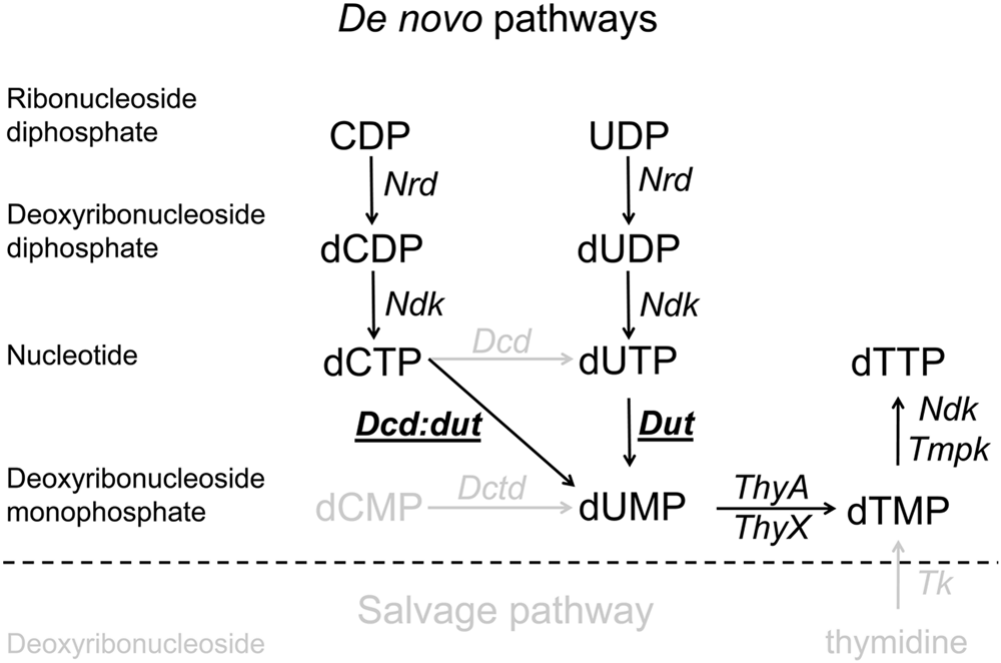
dTTP biosynthesis pathways and enzymes. Processes present in Mycobacteria are shown in black. Most organisms encode for additional de novo and salvage pathways that are shown in grey. Abbreviations: *Dcd* – dCTP deaminase, *Dut* – dUTPase, *Dctd* – dCMP deaminase, *Dcd:dut* – bifunctional dCTP deaminase: dUTPase, *Nrd* – Nucleoside diphosphate reductase, *Ndk* – Nucleoside diphosphate kinase, *Tmpk* – dTMP kinase, *Tk* – Thymidine kinase, *ThyA,ThyX* – thymidylate synthases.

Proper control of the intracellular concentration of deoxyribonucleoside-5-triphosphates (dNTPs), the building blocks of DNA, is critically important for efficient and high-fidelity DNA replication and genomic stability^1, 2^. Three of the four canonical dNTPs are synthesized from their respective ribonucleoside diphosphate (NDP) counterparts^3^. The direct precursor for dTTP, however, is missing from the ribonucleoside pool and is synthesized via separate routes (Fig. 1).

**Figure 2:**
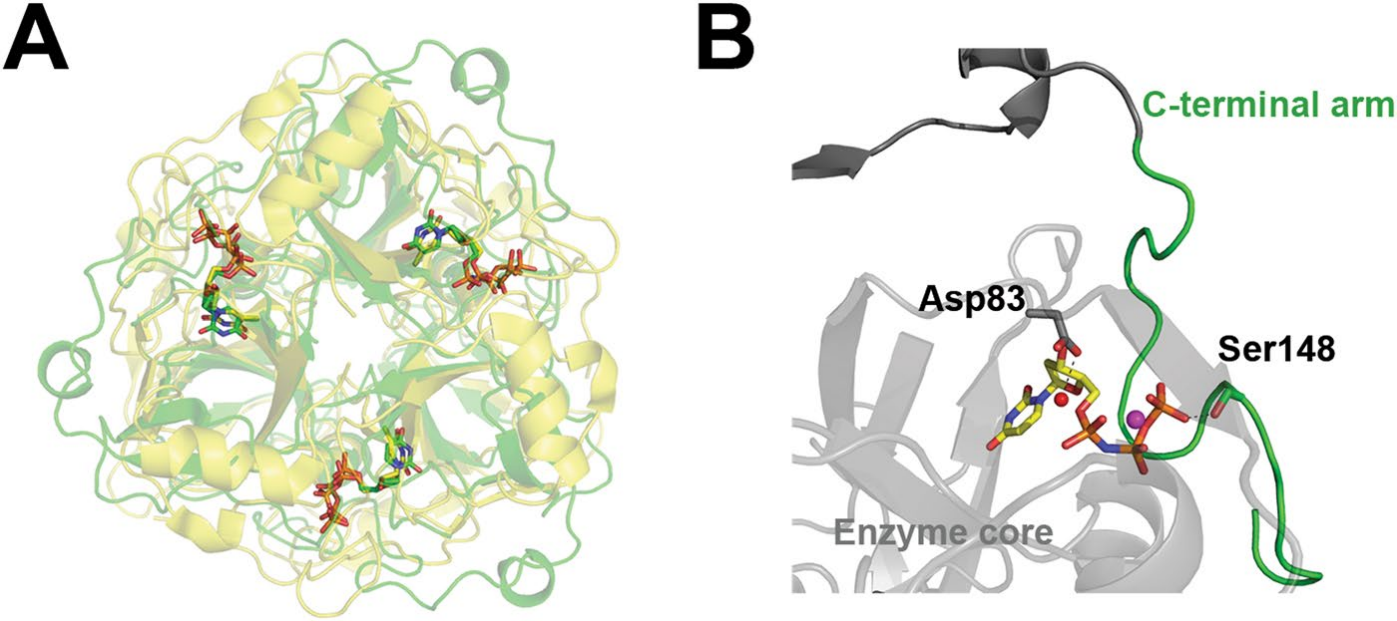
(**A**) Superposition of the quaternary structures of the *M. tuberculosis* dUTPase (Dut) depicted in green (PDB ID:2PY4) and the *M. tuberculosis* bifunctional dCTP deaminase/dUTPase enzymes (Dcd:dut) depicted in yellow (PDB ID:2QLP). Note the identical organization of the enzyme core of the homotrimers. Both structures contain the non-hydrolysable substrate analog α-β-imido-dUTP (dUPNPP) in the active sites. (**B**) Enlarged view of the active site of *M. tuberculosis* Dut showing the C-terminal arm in green. The side chains of the amino acids in case of point mutations and C-terminal arm truncation are shown with atomic colored stick representation similarly to the dUPNPP molecule and with green cartoon representation, respectively. The catalytic water is shown as a red sphere while the yellow sphere denotes the Mg^2+^ ion that coordinates the nucleotide.

The *de novo* synthesis of dTTP occurs through uracil base-containing precursors: dUMP is the direct input into the thymidylate synthase reaction (Fig. 1). In most organisms, the main dUMP supply is provided by the deamination of a cytosine deoxyribonucleotide (dCMP or dCTP) while other possible routes, e.g. the dephosphorylation of dUDP, are considered to be minor supplements^4–6^. When cytosine deamination occurs at the triphosphate level, the resulting dUTP is then converted into dUMP. The enzymes that catalyze these conversions belong to the dUTPase superfamily comprising dCTP deaminase (Dcd), dUTPase (Dut) and the bifunctional dCTP deaminase/dUTPase (Dcd:dut) (Fig. 1). These enzymes share the same quaternary structure as shown in Fig. 2A.

In addition to dUMP production, the dUTPase reaction also serves to eliminate excess dUTP to prevent uracil incorporation into DNA in place of thymine^7, 8^. Although not mutagenic when replacing thymine, the uracil in DNA is considered to be an error and induces uracil-excision repair mechanisms^9^. In high dUTP/dTTP ratios, however, DNA polymerases keep re-incorporating dUTP and the repair process becomes overwhelmed. Dut is ubiquitous and essential in most investigated cases^10–16^. Recently, novel functions of Dut emerged in gene expression regulation as well^15, 17–20^. Our genetic experiments also suggested that the mycobacterial Dut has a yet unknown but essential moonlighting function^11^.

In summary, Dut catalyzes the break-down of dUTP to dUMP and with this action it potentially takes part i) in dTTP biosynthesis, ii) in the maintenance of low dUTP/dTTP ratio to prevent uracil incorporation into DNA and iii) in interactions with regulatory proteins. The various roles now attributed to Dut and the large amount of knock-out and knock-down data on the dUTPase superfamily enzymes in various genetic backgrounds create a confusing picture of the contribution of Dut to the physiological processes in which it may be involved. As dTTP biosynthesis is an essential process and a major target in several current drug therapies, it is important to pinpoint those pathways in which Dut is a key contributing enzyme.

We therefore set-out to dissect the contributions of dUTP-hydrolyzing enzymes, Dut and Dcd:dut, to dTTP biosynthesis and to the prevention of DNA uracilation. For this reason, we searched for a simple model in which the obligatory dTTP precursor, dUMP, is produced exclusively by Dut and Dcd:dut in lack of salvage pathways and dCMP deamination (Fig. 1). This favorable set of conditions naturally occurs in the genus *Mycobacteria* ^11, 21^. Due to the exclusive biosynthetic role of Duts in these organisms, they present potential targets for drug development, as well. Earlier mutagenesis studies found the presence of the bifunctional Dcd:dut to be dispensable for growth in *M. tuberculosis* ^10, 22^ while the intact Dut protein is essential in *Mycobacteria* ^10, 11, 22^.

In the present study, we created *M. tuberculosis* Dut mutant proteins in which the enzyme activity is gradually tuned down. We then carried out genetic experiments in the fast-growing *M. smegmatis* in which we created the same Dut mutations and also included an inactive *dcd:dut* mutant strain in the experiments. We found that the dUTPase activity of either Dut or Dcd:dut can support cell growth. The double mutant *M. smegmatis* strain lacking the complete dUTPase activity, however, is inviable. We investigated the mutation-induced effects including dNTP pool changes, the mutation rate and the uracil content of DNA in *M. smegmatis* strains conferring various Dut and Dcd:dut mutants. Unexpectedly, the lack of Dut activity did not influence the biosynthesis of dTTP. We arrived to the conclusion that dTTP biosynthesis and the maintenance of genomic integrity by dUTP elimination are under differential control.

## Results

### Tuning down the activity of *M. tuberculosis* Dut

On the basis of our previous investigations on the human and *Escherichia coli* (*E. coli)* Duts^23, 24^, we planned and created three mutants of the *M. tuberculosis* Dut enzyme by site-directed mutagenesis. These mutants were chosen to represent enzymatic activity loss from one order of magnitude to the practical inactivity.

**Figure 3:**
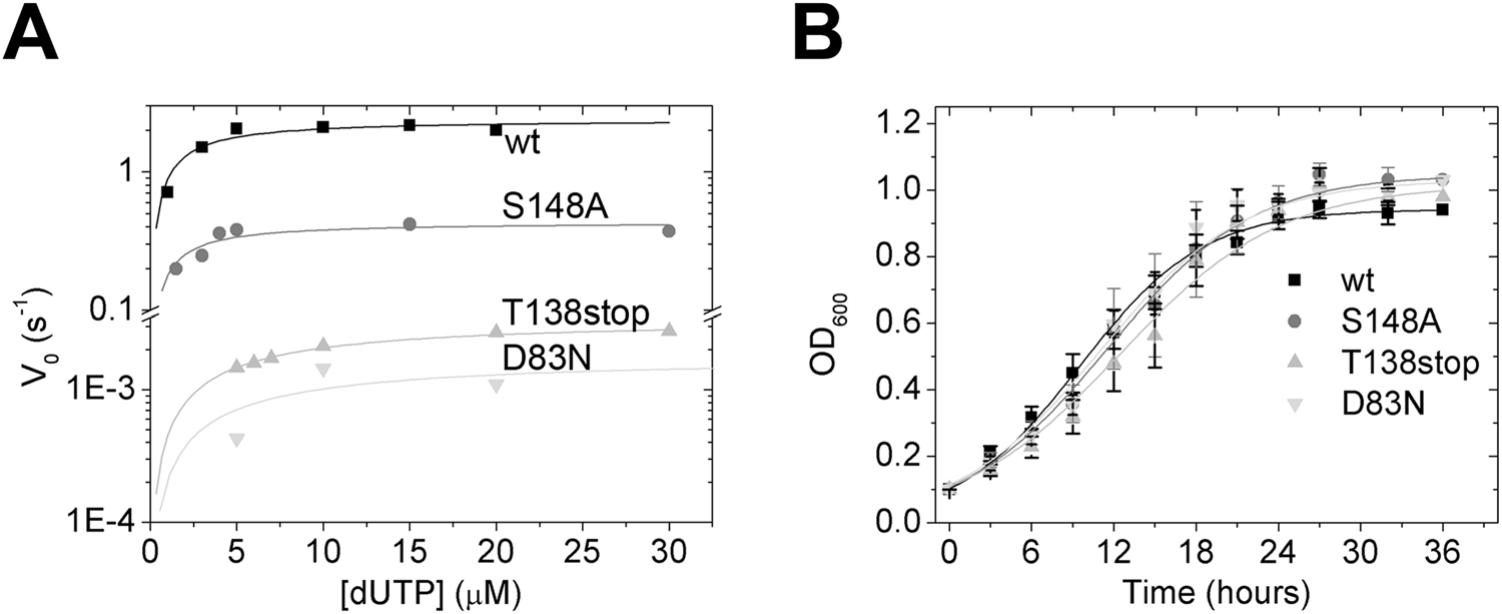
(**A**) Steady-state activity of wt and mutant Duts. Michaelis-Menten curves were measured using the phenol red pH indicator assay. Fitting the Michaelis-Menten equation to the curves yielded the following kcat and KM values: 1.22 ± 0.06 s^−1^ and 1.7 ± 0.5 μM for WT, 0.43 ± 0.04 s^−1^ and 1.5 ± 0.6 μM for S148A, 0.0035 ± 0.0001 s^−1^, 6.7 ± 0.4 μM for T138stop and 0.0013 ± 0.0005 s^−1^, 7.7 ± 6.7 μM for D83N mutant Dut. (**B**) *In vitro* growth analysis of wt and dut mutant *M. smegmatis* strains. The strains were grown in Lemco broth in shaking cultures for 2 days at 37 °C. Growth curves were prepared from (3*3) independent colonies from each mutation; means ± SD are plotted. Fitting the y = a/(1 + exp(−k*(x − xc))) equation to the curves yielded the following a, xc and k values: 0.94, 9.5 and 0.22 for WT, 1.04, 11.5 and 0.19 for S148A, 1.02, 12.4 and 0.17 for T138stop and 1.03, 11.0 and 0.19 for D83N Dut mutant strains.

The D83N substitution aims at compromising the coordination of the catalytic water by mutating the catalytic aspartate residue (Asp90 in *E. coli* Dut) (Fig. 2B). In effect, this mutant presents an extremely low catalytic activity (Fig. 3A, Table 1) similarly to what was observed in the *E. coli* enzyme^24^. The determination of the Michaelis constant (K_M_), however, is uncertain due to the limitations of the activity measurements at such low activities. The K_M_ could be better estimated in this case from the dissociation constant (K_d_) of the protein complexed with the non-hydrolysable substrate analog α, β-imido-dUTP (dUPNPP). We measured the K_d_ of the D83N.dUPNPP complex to be similar to that of the WT.dUPNPP complex (Table 1, Fig. S1B). Its catalytic efficiency being 0.0002 compared to the wt means that the D83N Dut is a practically inactive mutant.

**Table 1:** Kinetic parameters of the *M. tuberculosis* Dut and Dcd:dut enzymes.

Enzyme	k_cat_ (s^−1^)	K_M_ (μM)	K_d_.dUPNPP (μM)	k_cat_/K_M_ (M^−1^ s^−1^)	Efficiency
wt Dut	1.22 ± 0.06	1.7 ± 0.5	0.9 ± 0.5	7.18E + 05	1
S148A Dut	0.43 ± 0.04	1.5 ± 0.6	1.8 ± 1.0	2.87E + 05	0.4
T138stop Dut	0.0035 ± 0.0001	6.7 ± 0.4	3.9 ± 1.3	5.22E + 02	0.0007
D83N Dut	0.0013 ± 0.0005	7.7 ± 6.7*	1.5 ± 0.1	1.69E + 02*	0.0002
wt Dcd:dut dUTPase	0.033 ± 0.008	12 ± 3	—	2.75 E + 03	0.004
wt Dcd:dut dCTP deaminase	0.022 ± 0.005	20 ± 12		1.10 E + 03	NA
A115F Dcd:dut	no activity	—	—	—	—

*Data not reliable due to the limitations of the activity measurement.

NA: not applicable.

The efficient dUTPase catalysis requires conserved sequence motifs I-V from all three monomers of the Dut homotrimer^7^. Motifs I-IV constitute the active sites at the intermonomer clefts while motif V, located at the C-terminal arm, leaves the globular core of its monomer to associate with the neighboring active site and shield it from the solvent (C-terminal arm shown in green in Fig. 2B). This P-loop-like motif V changes conformation upon substrate binding and positions the phosphate chain of the nucleotide for efficient hydrolysis. The lack of this motif results in a nearly inactive enzyme in all investigated species^23, 25–33^. We created a mutant lacking conserved motif V by the truncation of the 154 amino acid long protein at position 138 (T138Stop). The T138Stop mutant exhibits 3-fold higher enzymatic activity than that of the D83N mutant the catalytic efficiency still being extremely low compared to the WT (Fig. 3A, Table 1). Both the K_d_ of the enzyme.dUPNPP complex (Fig. S1A) and the K_M_ are 4-fold higher than that of the WT complex (Table 1).

We created the S148A mutation to distort one of the hydrogen bonding interactions between the P-loop-like motif and the γ–phosphate of the substrate dUTP (Fig. 2B). The S148A mutant showed one order of magnitude loss in the enzyme activity (Fig. 3A, Table 1) similarly to an analogous mutant in the human Dut^23^. The K_M_, in concert with the K_d_, increased only 2-fold (Table 1, Fig. S1A).

### The A115F mutation inactivates the Dcd:dut enzyme

**Figure 4:**
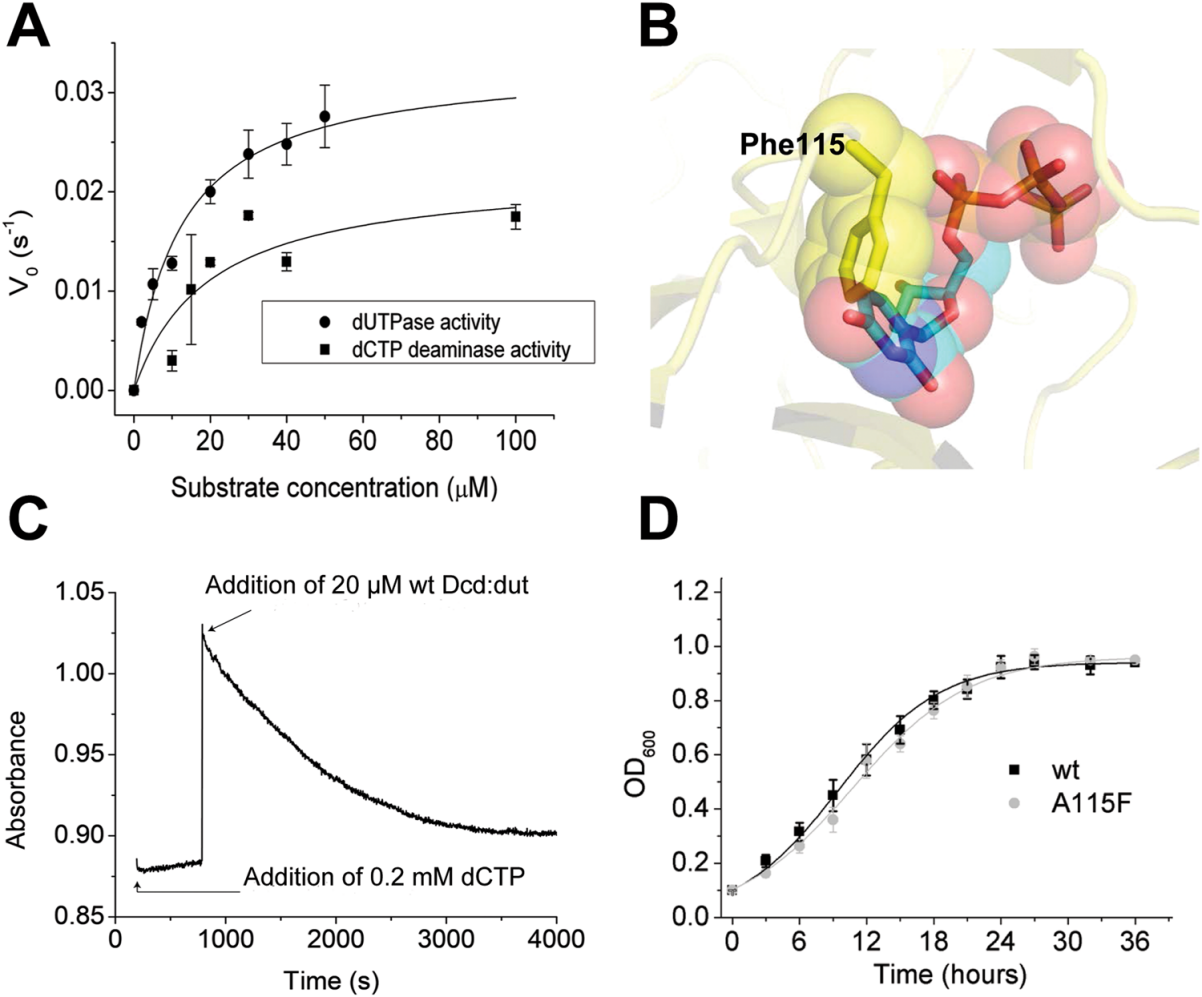
(**A**) Steady-state dUTPase and dCTP deaminase activity of wt Dcd:dut. The parameters yielded by fitting the Michaelis-Menten equation are shown in Table 1. The error represents the SD of 3 measurements. (**B**) The structural model of the active site of the *M. tuberculosis* Dcd:dut showing the steric conflict of the Phe115 side chain with the uracil ring of the substrate dUTP. (**C**) dCTP deaminase time course demonstrating the inactivity of the A115F Dcd:dut mutant. At t = 0, dCTP was added to a premix containing 0.02 mM A115F Dcd:dut. During ~500 s incubation time, no reaction (i.e. absorbance decrease) was detectable. The time course obtained upon the addition of the wt Dcd:dut enzyme confirmed that the assay was functional. (**D**) *In vitro* growth analysis of wt and Dcd:dut mutant *M. smegmatis* strains. The strains were grown in Lemco broth in shaking cultures for 2 days at 37 °C. Growth curves were prepared from (3*3) independent colonies from each mutation; means ± SD are plotted. Fitting the y = a/(1 + exp(−k*(x − xc))) equation to the curves yielded the following a, xc and k values: 0.94, 9.5 and 0.22 for the wt and 0.96, 10.8 and 0.19 for the A115F dcd:dut mutant strains.

We cloned and expressed the *M. tuberculosis* and *M. smegmatis* Dcd:dut enzymes and determined their steady-state kinetic parameters. In lack of significant difference between the behaviors of the *M. tuberculosis* and *M. smegmatis* Dcd:duts, we report the parameters for the *M. tuberculosis* enzyme for comparison with *M. tuberculosis* Dut constructs (Fig. 4A, Table 1). As expected^21^, the bifunctional enzyme is a relatively low-efficiency dUTPase compared to the monofunctional Dut (Table 1). In order to inactivate Dcd:dut without perturbing the overall structure of the enzyme, we introduced a bulky Phe into amino acid position 115 in place of an Ala (A115F) to prevent substrate binding to the active site. The Phe side chain in position 115 can only be accommodated within the active site cavity where it occupies the binding site of the uracil base (Fig. 4B). This mutant has the advantage that the cells keep synthetizing a structurally intact Dcd:dut protein. Fig. 4C shows that the Dcd:dut A115F does not exhibit enzyme activity. We have previously created this mutation in the structurally homologous human Dut and obtained an inactive but structurally intact mutant, as well^34^.

### The hydrolysis activity of either Dut or Dcd:dut supports the growth of *M. smegmatis*

We aimed to investigate the effect of dUTPase activity loss in the living cell. We therefore created the above mutations (S148A, T138Stop and D83N) within the genome of *M. smegmatis*. The *M. tuberculosis* and *M. smegmatis* Duts share 85% amino acid sequence identity (100% within the conserved motifs)^11^ and thus it is expected that the two enzymes behave similarly. In a previous paper, we established a method in which the disruption of the endogenous *dut* was rescued by a functional (complementing) copy of *dut* inserted into the genome on an integrating vector^11^. We used this scheme and introduced the mutations into the complementing copy of *M. smegmatis dut*. We obtained *M. smegmatis* strains that carried the mutant *dut* in a *dut* knock-out background (i.e. no wt copy present). Successful allele exchange was verified by Southern blot analysis (Fig. S2) and the mutations on the complementing *dut* copy were verified by sequencing of the appropriate genome region.

All three *dut* mutant strains were viable and unexpectedly, showed no growth defects when grown in liquid culture under stress-free conditions (Fig. 3B). This result suggests that the fully functional Dcd:dut in these *dut* mutant strains produces enough dUMP for the synthesis of more than limiting amounts of dTTP.

We also created a *M. smegmatis* strain carrying the non-functional A115F *dcd:dut* in a wt *dut* background. We constructed the A115F *dcd:dut* strain by allele exchange of the endogenous *dcd:dut* gene to a GFP-tagged copy carrying a point mutation. The A115F mutation was introduced either in wt or in inactive *dut* (D83N) background. Successful allele exchange was verified by Southern blot analysis (Fig. S2), and the mutation was verified by the sequencing of the appropriate genome region. The A115F *dcd:dut* strain encoding wt *dut* was viable and its growth rate was similar to that of the wt when grown in liquid culture (Fig. 4D). In contrast, we could not obtain any double mutant strains carrying both the *dut* D83N and the *dcd:dut* A115F mutations. In these cells, the dUTPase activity is completely abolished due to the fact that only inactive Duts are encoded. However, these inactive proteins are structurally intact and could still potentially mediate functions that are independent from their enzymatic activity or can operate in an inactive state (e.g. the essential surface loop in Dut^11^ and examples from other species^15, 18–20, 35^). This implies that the dUTPase activity is essential for viability and reinforces that the dUTPase activity has exclusive role in dTTP biosynthesis in *Mycobacteria* (cf. Fig. 1).

### Mutator phenotype of the dut and dcd:dut mutant strains

**Figure 5:**
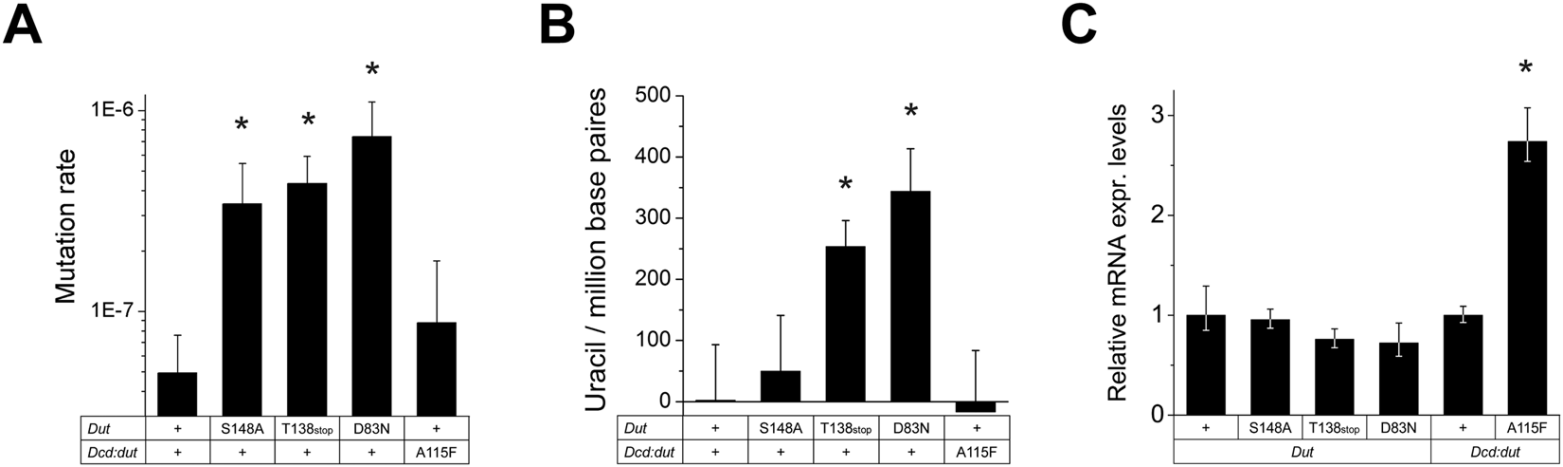
The effect of *dut* and *dcd:dut* mutations on the mutation rate, DNA uracilation and gene expression. (**A**) Mutation rates of mutant *M. smegmatis* strains. Means ± SE are calculated from (3*3) independent colonies from each mutation. Note that mutation rates of dut mutant strains directly correlate with the *in vitro* activity-loss of the corresponding mutant enzyme. Significance levels: P = 0.000115 for S148A, P = 0.000034 for T138stop and P =0.000004 for D83N. “+” denotes the wt enzyme. (**B**) Genomic uracil content of the mutant strains compared to the wt strain. Uracil contents were calculated from three independent strains from each mutant and normalized to the wt strain; means ± SE are plotted. Note that the genomic uracil content of dut mutant strains directly correlates with the *in vitro* activity-loss of the corresponding mutant enzyme. Significance levels: P = 0.074 for T138stop, P = 0.018 for D83N. (**C**) Quantitation of *dut* and *dcd:dut* expression levels in the wt and mutant *M. smegmatis* strains. mRNA levels were calculated from three independent strains from each mutant and normalized to their respective wt strain; means ± SE are plotted. Significance level: P = 0.029 for the A115F mutant.

As we could not reveal any obvious defects in the enzymatically compromised *dut* or *dcd:dut* mutant *M. smegmatis* strains, we investigated possible long term effects of these mutations. We measured the mutation rates in each of the mutant strains^36, 37^. We found that the mutation rates increased remarkably in the *dut* mutant strains (7-fold, 9-fold and 15-fold in the S148A, T138stop and D83N *dut* mutant strains, respectively). The mutation rate of the *dcd:dut* A115F strain appeared two-fold higher than the wt, however, the difference did not prove to be significant (Fig. 5A).

### Dut mutant strains accumulate uracil in their genome

We also measured the genomic uracil content of the mutant strains by a q-PCR based method developed in our laboratory^38^. This assay is based on the fact that the Pfu polymerase does not amplify uracil-containing template DNA while other polymerases do (e.g. Taq). It is therefore possible to calculate the relative uracil content of the sample on the basis of PCR efficiencies driven parallel by Pfu and by Taq polymerases. We found that *dut* mutants have elevated genomic uracil content compared to the wt strain and that the increase in uracil content correlates with the *in vitro* measured activity loss (50, 250 and 340 uracil/million base pairs in the S148A, T138stop and D83N *dut* mutant strains, respectively.) (Fig. 5B). For comparison, the 340 uracil/million base pairs genomic uracil content matches that measured in the *ung- E. coli* and the *ung-* MEF cells deficient in uracil misincorporation repair. The *dut-/ung- E. coli* strain contains even 20-times more uracils in its genome^38^. We could not detect any change in the genomic uracil content in the A115F *dcd:dut* mutant strain compared to the wt (Fig. 5B).

To investigate if these marked effects are not simply due to an underlying difference in gene expression, we measured the mRNA levels of the various constructs. The expression of the T138stop and D83N *dut* constructs in the *M. smegmatis* cells seems to decrease compared to the wt, however, the difference proved not to be significant (Fig. 5C). On the other hand, the expression level of the A115F *dcd:dut* construct increased significantly compared to the wt (Fig. 5C). This finding emphasizes the differential effects in DNA uracilation and mutagenicity between the *dut* and *dcd:dut* mutations even more.

### Pyrimidine nucleotide pool changes in the dut and dcd:dut mutant strains

**Figure 6:**
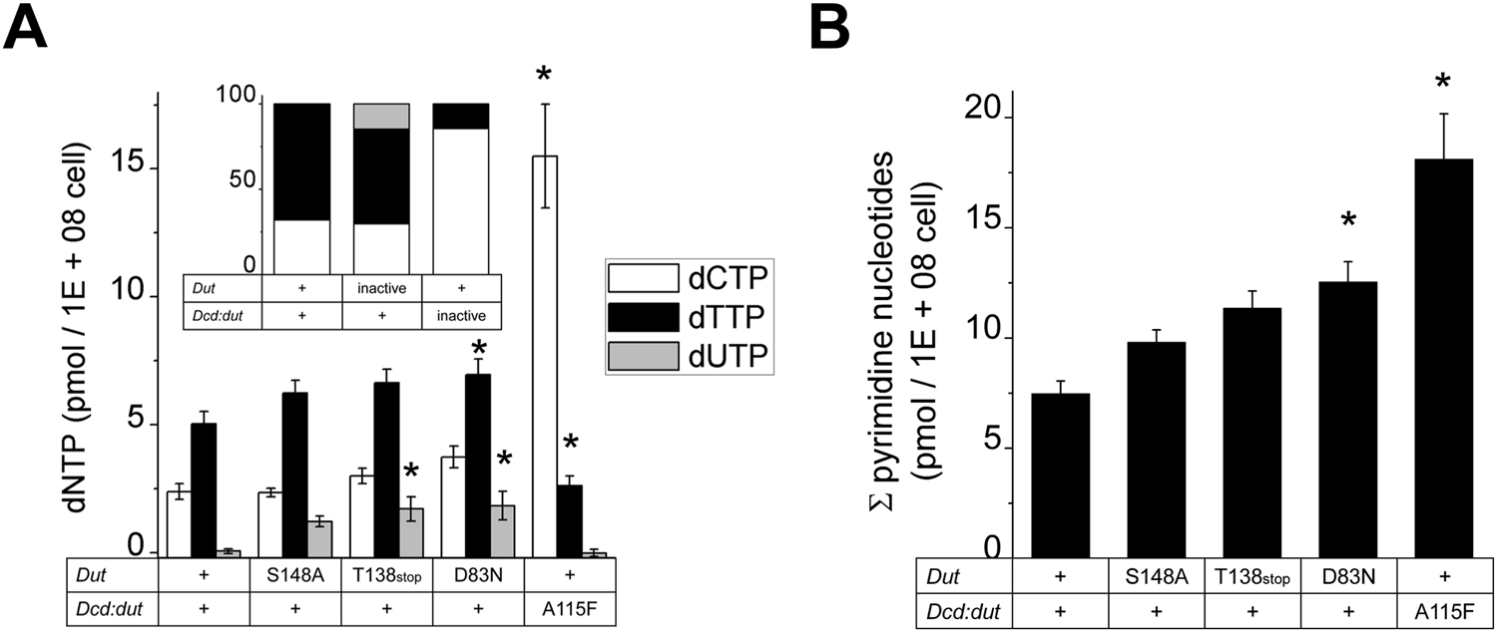
The effect of *dut* and *dcd:dut* mutations on the pyrimidine nucleotide pool. (**A**) dTTP, dCTP and dUTP concentrations were measured using a DNA polymerase-based method in all mutant strains. Mutant enzymes are indicated, “+” denote the wt enzyme. Means ± SE are calculated from 12 data points for each mutation. The dUTP concentration in the wt and in the A115F mutant falls out of the measurement range (<0.5 pmol/108 cells). Significance levels: P = 0.063 for the T138stop dUTP level, P = 0.095 for the D83N dTTP level, P = 0.047 for the D83N dUTP level, P = 0.000012 for the A115F dCTP level, P = 0.066 for the A115F dTTP level. The inset shows a comparison of the ratios of pyrimidine nucleotides within the wt and inactive mutant strains. (**B**) The change in total pyrimidine concentration in function of the mutation carried by each strain. Means ± SE are plotted. Significance levels: P = 0.05959 for D83N and P = 0.0028 for A115F.

To reveal the mechanism of uracil accumulation and mutation rate changes in our mutant strains, we measured the pyrimidine nucleotide pools in each strain. We used a DNA polymerase-based method to determine the concentration of dUTP, dTTP and dCTP in cell extracts^39, 40^. The method is based on the incorporation of radiolabeled dATP into a nucleotide-specific template limited by the concentration of the quantifiable dNTP. The concentration of dUTP in the wt strain proved to be too low to be accurately quantified in our assay (<0.5 pmol/10^8^ cells). We found that the concentration of dUTP became significantly elevated in the T138stop and D83N *dut* mutants (Fig. 6A). The dTTP:dUTP ratio changed from the >>10:1 ratio in the wt to 5:1 in the S148A *dut* mutant strain and to 4:1 in the T138stop and D83N *dut* mutant strains. The dTTP and dCTP concentrations remained quasi unchanged and consequently, the dTTP:dCTP ratio also remained in the wt range. However, the total pyrimidine content increased moderately but significantly in the D83N *dut* strain (Fig. 6, dCTP and dTTP concentrations increased 1.6- and 1.4-fold, respectively). Interestingly, however, the A115F *dcd:dut* strain had a normal low concentration of dUTP in its nucleotide pool while the dTTP:dCTP ratio became greatly imbalanced (Fig. 6 inset). The dCTP concentration of the A115F cell extract increased more than 6-fold while the dTTP concentration decreased to half of the wt (Fig. 6A).

These results clearly suggest two different roles for *dcd:dut* and *dut* in the regulation of pyrimidine nucleotide concentration and in the maintenance of genome integrity, respectively.

## Discussion

Previous knock-out studies found *dut* essential and *dcd:dut* dispensable for *Mycobacteria* ^10, 11, 22^. We reported that a complete knockout of *dut* is lethal^11^. We also reported that lethality is not due to the loss of the catalytic function but is mediated by a surface loop of unknown function independently of the dUTPase activity^11^. A well-folded and enzymatically active Dut enzyme lacking only this 5 amino acid long mycobacterium-specific surface loop does not support growth^11^. These previous studies did not provide information about the *in vivo* function of the enzymes bearing dUTPase activity, *dut* and *dcd:dut*. It was not clear, for example, how dUTPase activity affects the dNTP pool and downstream genomic processes. In the present study, we combined enzymology with genetics to address the role of dUTPase activity within the mycobacterial cell.

The mutant enzymes we created to tune down enzyme activity behaved in a well predictable manner in our *in vitro* experiments. The S148A and the T138stop *dut* mutations that compromised one or all interactions of the P-loop-like motif with the phosphate chain of the substrate resulted in small or severe activity loss, respectively, accompanied by weaker binding of the substrate (proportionally increased K_d_ and K_M_, Table 1). The D83N mutation impaired the coordination of the catalytic water which left the binding of the substrate unaffected (Table 1). However, the subsequent catalytic reaction was compromised resulting in practical inactivity (Table 1).

The activity-compromised *dut* point mutant *M. smegmatis* strains exhibit an increased mutation rate that accompanies the increase in the genomic uracil content (20–150 folds, Fig. 5). *Mycobacteria* encode three uracil DNA glycosylase enzymes (*ung, udgB and udgX*)^41–43^ enabling effective uracil excision repair in these organisms^42^. This functional redundancy reinforces that the accumulation of uracil in DNA is an unwanted process. The potential reason why uracil could still accumulate in the DNA of our *dut* mutants is that DNA polymerases re-incorporate uracil despite the excision repair mechanism constantly excising it from the mycobacterial DNA. Polymerases usually do not distinguish between dTTP and dUTP and thus, they will likely incorporate dUTP from a nucleotide pool containing highly elevated dUTP concentrations (Fig. 6). Uracils (U·A pairs) are generally not considered as mutagenic compounds^44^ while there is a controversy in the literature about the mutagenicity of abasic sites^44–46^. However, constantly excised and re-incorporated uracils probably cause stress and genome instability in the bacteria. In an *ndk, dut* double mutant *E. coli* strain, increased dUTP levels and replication intermediates from the uracil excision process caused several thousand-fold elevations in the mutation rate. The *Ung* mutation, which enables stable incorporation of uracil into DNA, could only partially alleviate the mutagenic effect^47^. This suggests that among other possible mechanisms, an increased frequency of uracil repair processed by either short patch or long patch base excision repair (BER) mechanism^48^ may be responsible for the observed elevation in the mutation rates. In addition, the *dut* mutation had a modest effect on dCTP and dTTP levels^47^. In our experiments, the most severe D83N *dut* mutation also resulted in a modest increase of cellular pyrimidine levels. The overproduction of dNTPs in response to endogenous DNA damage is a general stress response^3, 49, 50^ and most likely serves to promote tolerance against genotoxic stress. The elevated dNTP levels, in turn, increase the mutation rate^50–53^ as the activity balance of DNA polymerases is transiently altered allowing the proofreading activity to decrease for the benefit of the nucleotide incorporating function (i.e. next nucleotide effect)^52^. These considerations are in agreement with our observations as well. The equally large increase in the mutation rates of the various *dut* mutants also suggests that this phenomenon is part of a general stress response and not specific to dUTPase activity loss. Interestingly, although the dTTP:dCTP ratio was highly imbalanced in the *dcd:dut* mutant strain, it displayed only two-fold increase in the mutation rate possibly indicating that dCTP is a relatively poor mutagenic precursor (Figs 5, 6). These results are in good accordance with previous literature data also reporting 2-fold increase in the mutation rate using the rifampicin resistance method in *dcd* mutant *E. coli* ^46^. However, Schaaper and Matthews also found that the experimental system greatly affects the observation of mutagenicity. In their study, the 2-fold increase in the mutation rate observed using the rifampicin resistance assay appeared much higher (<42-fold) using a different assay (only available for *E. coli* for the moment)^46^. Moreover, Kumar and his colleagues found that even mild dNTP pool imbalances were mutagenic in *Saccharomyces cerevisiae*. However, the mutagenic potential of different imbalances did not directly correlate with their extent^53^. Nordman and Wright proposed that dNTP imbalances were not responsible for increased mutation rate in the *ndk, dut* mutant *E. coli* ^47^. Our findings suggest that the dCTP:dTTP imbalance results in lower mutagenic potential than that of DNA uracilation.

Unexpectedly, the reduced dTTP concentration in the *dcd:dut* mutant strain did not limit the proliferation rate of the bacteria (Fig. 4D). This indicates that the activity of either of the two dUTPases is sufficient to support dTTP synthesis for the efficient growth of *M. smegmatis*. Our further results shown in Fig. 6 suggest, however, that dUMP production and dTTP biosynthesis are mainly under the control of the bifunctional Dcd:dut enzyme. The fact that Dcd:dut is a hundred-fold less efficient dUTPase than Dut (Table 1 and ref 21, 54) suggests that the mechanistic differences between the two dUTPase enzymes are more important than simply is their catalytic efficiency. Dcd:dut is able to bind dTTP which inhibits its activity^55^. This negative feedback inhibition allows for the regulation of the cellular dCTP:dTTP concentration ratio. Dut, however, can only accommodate dUTP and does not show any allosteric features^56^. Based on our results shown in Figs 5, 6, we propose that Dut is responsible for the efficient elimination of dUTP, while the role of the bifunctional Dcd:dut is to maintain the proper dCTP:dTTP ratio. There are clear advantages to such functional diversion. dUTP is constantly generated from dUDP by the nucleoside diphosphate kinase (*Ndk*) and also by spontaneous dCTP deamination in mycobacteria (and by other enzymatic pathways in eukaryotes and in some prokaryotes (Table 2). The accumulation of dUTP is efficiently prevented by the monofunctional Dut. The purpose here is to sanitize the dNTP pool from dUTP to prevent genome uracilation. The regulatory capabilities are built in the other, closely related, Dcd:dut enzyme that proved to be an inefficient dUTP sanitizer but keeps the pyrimidine DNA building blocks in a correct concentration ratio (Fig. 6). The preventive aspect of the bifunctional enzyme may be that dangerous dUTP is not released following the dCTP deamination reaction but is converted to dUMP on the same enzyme^21, 57^.

**Table 2:** The effects of dUTPase modification in various organisms.

Organism	Pathways	Phenotype	Rescue	dNTP pool/Mutagenicity	Ref.
*E. coli*	Dcd, Dut, Tk	KO lethal; Mutants: thymidine auxotroph, filamentous, hyperrec (nicks in DNA), prolonged generation time, increased sensitivity to 5′- FUs	Dcd-, ung- rescues synthetic lethality with pyrE, xth, recA, recBC but not with Tk	dUTP 10x up, dTTP 3x up, mutagenicity 5–15x up	12, 59, 68–70
*M. smegmatis*	Dcd:dut, Dut	Dut KO lethal; Mutants: normal generation time, high genomic U content	No rescue with active but loop(−) Dut	dUTP 20x up, mutagenicity ~15 × up	11, this study
*T. brucei*	Dut, Tk	KO lethal; Mutants: cell cycle alterations, chromosome fragmentation, sensitive to MTX	Thymidine supply (ung- increases cytotoxicity)	dUTP 9x up, mutagenicity 9x up	13, 71, 72
*C. elegans*	Dctd, Dut, Tk	embrionic lethality; ATL1, RAD51 foci –> S-phase checkpoint activation	ung1, clk2 rescues, thymidine supplemented medium only partially	n.d.	14
*D. melanogaster*	Dctd, Dut, Tk?	lethality in early pupal stage, DNA strand breaks, U in DNA	n.d.	n.d.	15
*S. cerevisiae*	Dctd, Dut	dTMP auxotrophs (no Tk!), growth delay, cell cycle abnormalities	exogenous dTMP, ung inactivation rescues, APE inactivation does not	mostly AT –> CG mutations	58, 73
*Human cell lines*	Dctd, Dut, Tk	sensitization for FdUrd, even more with Tmk double silencing; decline in clonogenic survival, increase in DNA double strand breaks and in Tmk, Tk expression levels; genome instability, tumorigenesis; apoptosis in pancreatic beta cells	n.d.	variable, no significant change or n.d.	67, 74–77
*Arabidopsis*	Dctd, Dut, Tk	lethality or sterility, sensitive to 5FUs, 7-fold increase in homologous recombination events	critical partners unknown	n.d.	16, 78

We compiled the available literature data on the *in vivo* effects of dUTPase activity loss in various organisms in Table 2. This comprehensive data set supports a similar partition between the dUTP eliminating and the dNTP balancing functions in other organisms that bear additional dTTP producing pathways, as well. In *Saccharomyces cerevisiae* and *Caenorhabditis elegans* (bearing dCMP deaminase and a salvage pathway), the inactivation of *dut* is lethal. The inhibition of *ung*, however, can rescue the observed phenotype in these organisms^14, 58^ indicating the deleterious effect of genome uracilation in the *dut* mutant. The numerous reports on *E. coli dut* mutants also indicate that the major effect of Dut inactivation is genome instability and not a short supply of dTTP (Table 2). While the *dut*-1/*ung*-1 mutant *E. coli* strain can be maintained, the *dut-1* phenotype is lethal^59^. However, in *Trypanosoma brucei*, in which there is no dCTP/dCMP deaminase and dUMP production strongly depends on dUDP/dUTP hydrolysis, the inactivation of *ung* could not rescue *dut* silencing but instead increased the cytotoxic effects conveyed by the low dUMP levels^13^. Efficient *dut* silencing in human cell lines resulted in a decline in clonogenic survival due to genome instability (Table 2). All these literature data support the major role of *dut* in dNTP sanitizing. The regulation of dTTP concentration, however, seems to be exclusively committed to dCTP and/or dCMP transforming enzymes (Dcd, Dcd:dut, Dctd) which may be structurally unrelated from each other but are all allosterically regulated^55, 57, 60–62^. A further in-depth investigation of the de-coupling of these functions may shine light on mechanisms supporting the appearance of T in and the exclusion of U from DNA.

## Methods

### Bacterial strains, media and growth conditions

*M. smegmatis mc* ^*2*^ *155* was grown in Lemco medium (broth) or in Lemco with the addition of 15 g L^−1^ Bacto agar (solid). Kanamycin was added at 20 μg/ml, hygromycin B at 100 μg/ml, gentamicin at 10 μg/ml, and streptomycin at 20 μg/ml final concentration. For sucrose selection, 5% (wt/v) sucrose was included in the medium. X-Gal (5-bromo-4-chloro-3-indolyl-b- D- galactopyranoside) was used at 40 μg/ml.

### Mutagenesis, cloning and gene expression

All recombinant proteins were expressed in *E. coli* BL21(DE3)pLysS cells using the *M. tuberculosis dut* gene (Rv2697c) and the *M. smegmatis* and *M. tuberculosis dcd:dut* genes (MSMEG_0678 and Rv0321 respectively). The *M. tuberculosis* pTBdcd7 Dcd:dut expression plasmid was kindly provided by Martin Willemoes. The site-directed mutagenesis of Dut was carried out according to the Stratagene QuikChange site-directed mutagenesis instructions and verified by sequencing of both strands. The recombinant Dut carrying an N-terminal hexa-His tag was cloned into pET19-b vector, and the recombinant *M. smegmatis* Dcd:dut amplified from p2NIL_dcdWT and p2NIL_dcdA115F (created in this study) was cloned into pET45-b vector with restriction sites BamHI and HindIII (Table S1). Both proteins were expressed in *E. coli* BL21(DE3)pLysS cells. For protein overexpression, the cells were grown to an OD_600_ of 0.4, treated with 0.5 mM isopropyl-b-D-thiogalactopyranoside (IPTG) at 37 °C for 3 hours for Dut and at 30 °C for 6 hours for Dcd:dut expression.

### Protein purification

Pellets of cells expressing Dut were lysed in a buffer containing 50 mM TRIS pH 7.5, 100 mM NaCl, 0.5 mM EDTA, 1 mM DTT, 0.1 mM PMSF and EDTA-free protease inhibitor (Roche). Dcd:dut expressing cells were lysed in a buffer containing 20 mM HEPES pH 7.5, 100 mM NaCl, 5 mM MgCl_2_, 10 mM ß-ME, 0.1% v/v TRITON-X-100, ca. 10 μg/ml RNase, ca. 100 μg/ml DNase, 5 mM benzamidine, 0.1 mg/ml lysozyme and EDTA-free protease inhibitor (Roche). Cell suspensions were sonicated (3 × 60 s) and centrifuged (15550g, 30 min). The final Dut and *M. smegmatis* Dcd:dut supernatant after cell extraction was loaded onto a Ni-NTA column (Novagen) and purified according to the Novagen protocol. The *M. tuberculosis* Dcd:dut was purified on Q-Sepharose (GE Healthcare) anion-exchange column, followed by gel filtration on a Superdex 75 column (GE Healthcare) using an AKTA Explorer purifier. The purity of the protein preparation was analyzed by SDS-PAGE. Protein concentration was measured using the Bradford method (Bio-Rad Protein Assay) and by UV absorbance (λ_280_ = 8480 M^−1^ cm^−1^ for H145W *M. tuberculosis* Dut and its mutant enzymes, and λ_280_ = 9970 M^−1^ cm^−1^ for the *M. smegmatis* Dcd:dut and the A115F mutant enzyme, λ_280_ = 11460 M^−1^ cm^−1^ for the *M. tuberculosis* Dcd:dut and the A115F mutant enzyme) and is given in monomers.

### Steady-state colorimetric dUTPase assay

Protons released in the dUTPase reaction were detected by phenol red pH indicator in 1 mM HEPES pH 7.5 buffer also containing 100 mM KCl, 40 μM phenol red (Merck) and 5 mM MgCl_2_. A Specord 200 (Analytic Jena, Germany) spectrophotometer and 10 mm path length thermostatted cuvettes were used at 20 ^o^C for measuring dUTPase activity of the wt and mutant enzymes. The absorbance was recorded at 559 nm. V_0_ was extracted from the raw absorbance vs. time curves followed by fitting the Michaelis-Menten equation to the V_0_ vs. substrate concentration steady-state curves using Origin 7.5 (OriginLab Corp., Northampton, MA).

### dCTP deaminase activity measurements

The dCTP deaminase activity was measured in a continuous spectrophotometric assay using the difference in the molar extinction coefficients between deoxycytidine and deoxyuridine (Δε_286_ = 3240 M^−1^ cm^−1^). The absorbance was recorded at 286 nm. The assay was buffered with 20 mM HEPES pH 7.5 also containing 100 mM NaCl and 5 mM MgCl_2_. The reaction was initiated by the addition of deoxicitidine triphosphate into the enzyme containing premix. A Specord 200 (Analytic Jena, Germany) spectrophotometer and 10 mm path length thermostatted quartz cuvettes were used at 20 °C. V_0_ was extracted from the row absorbance vs. time curves followed by fitting the Michaelis-Menten equation to the V_0_ vs. substrate concentration steady-state curves using Origin 7.5 (OriginLab Corp., Northampton, MA).

### Construction of the *dut* mutant strains

All genetic experiments were carried out in *M. smegmatis mc* ^*2*^ *155* using *M. smegmatis* genes (MSMEG_2765 *dut* and MSMEG_0678 *dcd:dut*) for complementation. *Dut* KO SCO cells were used^11^ to construct our *dut* mutant strains. The mutant *dut* containing complementing vectors were created by the QuikChange method (Stratagene) using the vector pGem-dut^11^ as template. Mutant strains were constructed by electroporating the *Dut* KO SCO strains with the appropriate complementing plasmids. Double crossovers (DCOs) carrying the mutant *dut*s were selected by colony PCR (Table S1) and verified by Southern blot (Fig. S2) and by sequencing the appropriate genome region. Three parallel strains from each mutant were chosen and used for forward experiments. Primers used for cloning, mutagenesis and screening are compiled in Table S1.

### Construction of the *dcd:dut* mutant strains

*Dut* KO strains carrying the wt^11^ or the D83N mutant complementing *dut* copy were used to construct the A115F *dcd:dut* mutant and the D83N *dut*/A115F *dcd:dut* double mutant strains, respectively. A 3.5 kb fragment containing the *dcd:dut* gene and its flanking regions was cloned into p2NIL using HindIII restriction sites (Table S1) generating p2NIL_dcdWT. The A115F mutant *dcd:dut* containing vector was created by a modified QuikChange method^63^. A green fluorescent protein from pLL192^64^ was C-terminally fused to the *dcd:dut*. The 6.1 kb PacI cassette carrying the *lacZ* and *sacB* selection markers from pGOAL17 was cloned into the sole PacI site of p2NIL to yield p2NIL_dcdA115F. p2NIL_dcdA115F was electroporated into electrocompetent cells. DCOs carrying the mutant *dcd:dut* were selected by colony PCR and verified by Southern blot (Fig. S2), then finally by sequencing the appropriate genome region. Three parallel strains were chosen and used for forward experiments. While the *dcd:dut* allele exchange worked in the wt *dut* background, it did not work in the D83N *dut* background even after several trials. Primers used for cloning, mutagenesis and screening are compiled in Table S1.

### Growth assays

*M. smegmatis* mutant strains were grown in liquid media. OD_600_ was measured every 3 hours. Three parallel strains were used from each investigated strain (9 parallel from each mutation) in these experiments. For quantitative comparison, growth curves were fitted with the y = a/(1 + exp(−k*(x − x_c_))) equation that yielded the best fit keeping the function as simple as possible.

### Determination of the spontaneous mutation rate

To determine the spontaneous mutation rates, three rifampicin sensitive independent colonies of each of the three strains/applied mutations were used to inoculate cultures that were grown at 37 °C, 150 rpm. Saturated cultures were serially diluted in sterile broth and plated onto agar plates to determine total CFUs or onto agar plates supplemented with 100 µg/ml rifampicin. The mutation rate for each of the 9 (3 × 3) independent cultures/applied mutation was determined as follows,

$${\rm{\mu }}=[({{\rm{m}}}_{{\rm{t}}}{/N}_{{\rm{t}}})-({{\rm{m}}}_{{\rm{0}}}{/N}_{{\rm{0}}})]\times \,\mathrm{ln}({{\rm{N}}}_{{\rm{t}}}{/N}_{{\rm{0}}})$$

where m_0_ is the observed number of mutants at time point 0, m_t_ is the observed number of mutants at the next time point, and N_0_ and N_t_ are the numbers of cells at time points 0 and t, respectively. The mean mutation rate was calculated for each mutant^36^.

### Genomic DNA isolation

10 ml liquid culture was grown until OD_600_ = 0.5 and harvested. The cells were resuspended in 1 ml 10 mM Tris, pH 7.5 and 0.1 mm glass beads were added to 2 ml volume. The cells were disrupted by vortex and incubation on ice by turn. After centrifugation, the supernatant was manipulated routinely to purify DNA by phenol:chloroform:IAA (25:24:1) extraction followed by isopropanol precipitation.

### Determination of the genomic uracil content

In order to quantify the uracil content of DNA, a real-time quantitative PCR-based assay was used^38^. Genomic DNA was isolated and digested with BamHI. DNA fragments of 5 kb were purified from gel. Real-time PCR was performed on a Mx3000P qPCR System (Agilent Technologies) using EvaGreen dye (Biotium) and PfuTurbo Hotstart DNA polymerase (Stratagene) and Mytaq Hotstart DNA polymerase (Bioline). A segment with 1017 base length defined by the primers (Table S41) was amplified during the PCR reaction. Two-fold dilution series were prepared from the DNA samples. Three parallel strains were used for each mutation in the experiments.

### dNTP extraction

Exponential phase cells were grown with appropriate antibiotics until OD_600_ = 0.6. The total CFUs were determined for each culture, and cells were centrifuged for extraction. Washed pellets were extracted in 0.5 ml ice-cold 60% methanol overnight at −20 °C. Cells were removed by centrifugation (15–20 min, 13.000 rpm) the methanolic supernatant was boiled for 5 min and centrifuged. The supernatant containing the soluble dNTP fraction was vacuum-dried (Eppendorf) at 45 °C, 1h. Extracted dNTPs were dissolved in 50 μl dUTPase buffer (30 mM Tris-HCL, pH 7.5, 10 mM MgCl_2_, 50 mM NaCl, 1 mM EDTA) and stored at −80 °C.

### The quantitation of *dcd:dut* and *dut* expression levels

Cells were grown in 50 ml liquid culture until saturation, washed in ice cold PBS and harvested by centrifugation (3100 g, 20 min). Bacterial pellets were resuspended in 1 ml Trizol (Life Technologies), and the cell wall was disrupted by repetitive vortexing with glass beads (6 × 1min). Nucleic acid recovered in the aqueous phase after addition of 0.2 ml chloroform was precipitated with the addition of 0.5 ml isopropanol. The RNA preparations were DNase-treated (10 min, 37 ^o^C) and purified with the Nucleospin RNA Clean-up kit according to the instructions of the manufacturer. Mycobacterial RNA yield were assayed using the Nano-Drop ND-2000 Spectrophotometer (NanoDrop Technologies). RNA samples were amplified from 1 µg total RNA by random hexamer primers using the Transcriptor First Strand cDNA Synthesis Kit (Roche). The resulting cDNA was quantified by Quantitative PCR using EvaGreen (Bioline) and MyTaq PCR master mix (Bioline) in a Stratagene Mx3000P instrument. *sigA* (MSMEG_2758), an endogenous reference gene was used to normalize input cDNA concentration^65^. The relative expression ratios of the examined genes were calculated using the comparative Ct method (ΔΔCt). Primers used to measure cDNA of *sigA, dcd* and *dut* are compiled in Table S1.

### Determination of the pyrimidine nucleotide pool size

The determination of the pyrimidine nucleotide pool size in each extract was based on DNA polymerase-catalyzed incorporation of radioactive dNTP into the synthetic oligonucleotide template method described in ref. 66. The reaction mixture (50 μl) contained *Klenow* buffer, 0.5 unit exonuclease negative *Klenow-*fragment (Fermentas), 0.25 μM dTTP/dCTP specific template, 0.25 μM primer (Table S1), 2.5 μM [3H] dATP (1,5 Ci/mmol) (American Radiolabeled Chemicals, Inc.) and 8 μl dNTP-extract or premixed dNTP for calibration. Calibration curve was prepared using 0, 0.1, 0.5, 1, 2, 4 and 8 pmol of each dNTPs/reaction mixture. Incubation was carried out for 60 min at 37 °C and the reaction mix was spotted onto DE81 paper. The papers were dried, washed (3 × 10 min) with 5% Na_2_HPO_4_, and rinsed once with distilled water and once with 95% ethanol. After drying, radioactivity on the papers was measured in a liquid scintillation counter (Beckman). In case of the dCTP measurement, we used Taq polymerase (RedTaq, Sigma), the incubation was carried out at 48 °C for 1 hour, as Klenow polymerase is capable of incorporating CTP and GTP from nucleotide extracts^39^.

dUTP concentration was measured according to Koehler *et al*.^67^. Half of the samples for dTTP measurement were treated with 40 ng recombinant Dut at 37 °C, 45 min. The Dut enzyme was precipitated with 60% methanol. The dUTP in half of the extract was enzymatically hydrolyzed while in the parallel sample it was not. Then dUTP concentration was measured using the same protocol as for dTTP determination.

Two-fold dilution series were prepared from cell extracts in the polymerase reactions. Four samples for each of the three parallel strains were used for each mutation in the experiments (i.e. 12 data points).

### Statistical analysis

Statistical analysis was carried out using the STATISTICA.13 software. The non-parametric Kruskal–Wallis test or the one-way ANOVA test with Student-Newman-Keuls multiple comparison post-hoc test was used when samples passed the equal variance (Bartlett’s) criterion.

## Electronic supplementary material

Supplementary Information
