# Supplementary material for: Differential control of dNTP biosynthesis and genome integrity maintenance by the dUTPase superfamily enzymes
Source: Sci Rep. 2017 Jul 20;7:6043. doi: 10.1038/s41598-017-06206-y (PMC5519681; doi:10.1038/s41598-017-06206-y)
Supplement: Supplementary file 1 — Supplementary Information [file 41598_2017_6206_MOESM1_ESM.pdf]

Supplementary Information

Differential control of dNTP biosynthesis and genome integrity maintenance by the dUTPase superfamily enzymes

Rita Hirmondó*[1], Anna Lopata*[1], Éva Viola Surányi*[1,2], Beáta G. Vértessy[1,2] and Judit Tóth[#1]

1 Institute of Enzymology, RCNS, Hungarian Academy of Sciences, Budapest, Hungary,

2 Dept of Applied Biotechnology, Budapest University of Technology and Economics, Budapest, Hungary

*equal contribution

[#] Corresponding author:

Judit Toth

Institute of Enzymology, Res Cent for Nat Sci, HAS

Magyar tudósok körútja 2, H-1117, Budapest, Hungary

Phone: +36 1 382 6707

e-mail: toth.judit@ttk.mta.hu

Figure S1 Effect of the created mutations on the substrate binding of *M. tuberculosis* dUTPase (**A**) CD equilibrium titrations. Comparison of ligand (dUPNPP) binding to the WT, S148A and to the T138stop mutant dUTPase. Solid lines represent quadratic fits to the data yielding the following K_d values: 0.9 ± 0.5 μM for the wt, 1.8 ± 1.0 μM for the S148A and 3.9 ± 1.3 μM for the T138stop mutant. (**B**) Fluorescence intensity titration of the single Trp in the D83N mutant upon dUPNPP binding. The smooth line through the data is a quadratic fit yielding the K_d value of 1.5 ± 0.6 μM. Errors represent S.D. for n = 3. For more parameters see Table 1.

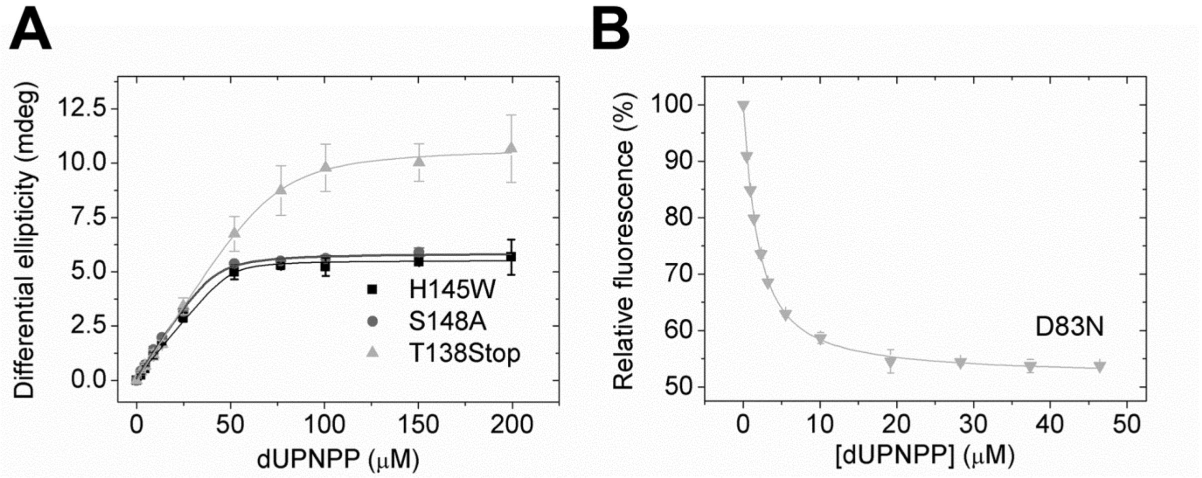

Materials and Methods

Fluorescence intensity titrations. Fluorescence was measured in a Jobin Yvon Spex Fluoromax-3 spectrofluorometer at 20 °C, with excitation of the single Trp at 295 nm (slit 1 nm) and emission at 347 nm (slit 5 nm). 4 μM protein was titrated by the addition of 1-2 μl aliquots from concentrated dUPNPP solutions (purchased from Jena Bioscience, Germany). Because large concentrations of nucleotides were used, care was taken to correct for any additional fluorescence or inner filter effect imposed on the measured intensities by the nucleotide stock solutions.

Circular dichroism intensity titrations. CD spectra were recorded at 20 °C on a JASCO 720 spectropolarimeter using a 10 mm path length cuvette. 50 μM protein was titrated by stepwise addition of the non-hydrolysable substrate analogue dUPNPP, in a buffer containing 20mM HEPES pH 7.5, 50mM NaCl and 2 mM $MgCl_2$. A spectrum between λ = 240-350 nm was recorded at each nucleotide concentration. Differential curves were obtained by subtracting the signal of dUPNPP alone from that of the corresponding complex. Differential ellipticity at λ_{max} = 269 nm was plotted against the

dUPNPP concentration to obtain the binding curves. The following quadratic equation was fitted to the experimental curves:

$$y = s + A * \left((c + x + K) - \sqrt{(c + x + K)^2 - 4 * c * x} \right) / 2 * c$$

s = y at x = 0; A = amplitude; c = protein concentration; K = K_d

Figure S2 Southern blot analysis of the mutant strains

Southern blot analysis resulted in 1.5 kb and 3.3 kb bands in the case of wt *dut* and the S148A, T138stop and D83N mutant *dut* strains, and 5.4 kb and a 2.1 kb bands in the case of wt *dcd:dut* and A115F *dcd:dut* mutant strains, respectively.

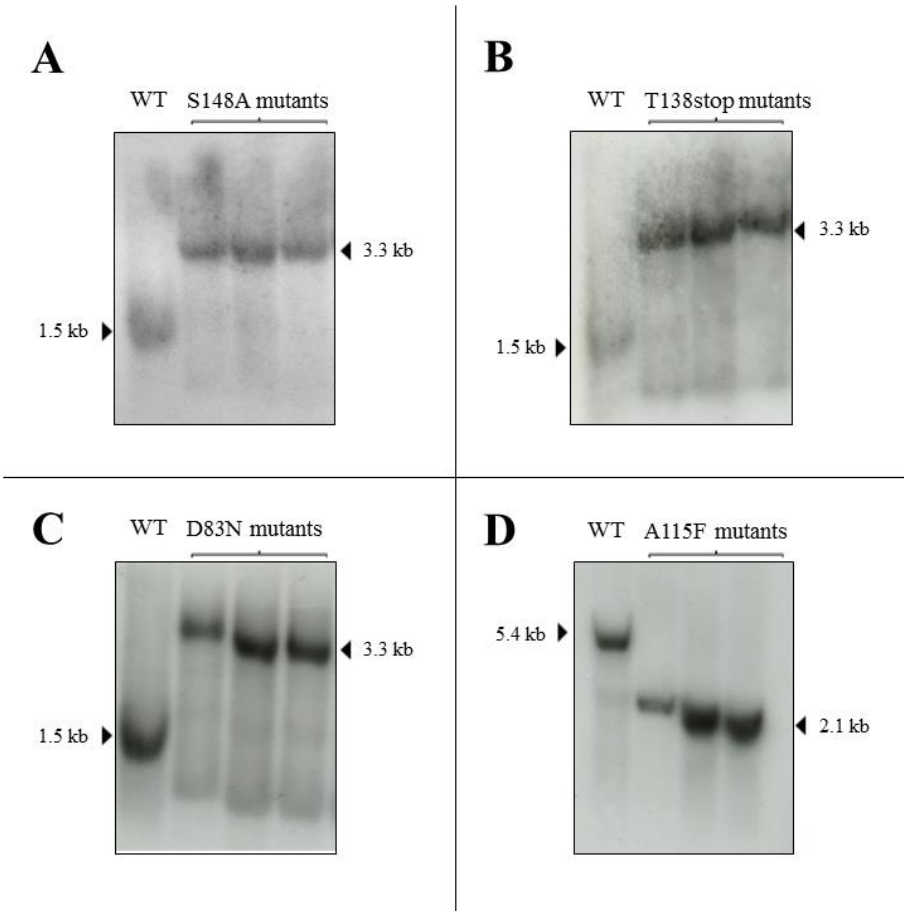

Materials and Methods

Southern-blot analysis. Southern-blot analysis was carried out using the DecaLabel TM DNA Labeling Kit (Fermentas) according to the manufacturer's instruction. Restriction digestion of the genomic DNA was performed using NcoI and PstI resulting in 1.5 kb and 3.3 kb fragments in the case of WT and *dut*-disrupted mutant strains, respectively. The probe was a 0.7 kb fragment encompassing the *dut* gene (for primers see Table S2). Restriction digestion of the genomic DNA was performed using NcoI resulting in 5.4 kb and 2.1 kb fragments in the case of WT and A115F *dcd:dut* mutant strains, respectively. The probe was a 0.5 kb fragment following the coding region of the *dcd:dut* gene (for primers see Table S2).

Table S1. Oligonucleotide sequences used in this study

Primer sequence	Application	
5’- GGTGGCTGGGGTTCCGCCGGCGGACATGCG -3’	S148A mutant	QuikChange mutagenesis to create mutant Mtb dut enzymes
5’- CGCATGTCCGCCGGCGGAACCCCAGCCACC -3’		
5’- GGGCTGGCCTCGTGATCCCGCGGCGACGG -3’	T138stop mutant	
5’- CCGTCGCCGCGGGATCACGAGGCCAGCCC -3’		
5’- CCGGGCACCATCAACGCGGGTTATCGTGGGG -3’	D83N mutant	
5’- GGCCCGTGGTAGTTGCGCCCAATAGCACCCC -3’		
5’- CGGTCACGGTTCCGCCGGCGGACATGCG -3’	S148A mutant	QuikChange mutagenesis to create complementing *dut* mutant pGem vectors
5’- CGCATGTCCGCCGGCGGAACCGTGACCG -3’		
5’- GCCGGTTTGGCGGACTGAACCCGTGGCG -3'	T138stop mutant	
5’- CGCCACGGGTTCAGTCCGCCAAACCGGC -3'		
5’- CCGGCACGATCAACGCCGGCTACCG -3'	D83N mutant	
5’- CGGTAGCCGGCGTTGATCGTGCCGG -3'		
5’- CAACCTGGATCCGCAGACG -3'	SCO, DCO screening for *dut* mutant *M. smegmatis* strains	
5’- CACCTTCCTGCACGACTTCG -3'		
5’- CGTCTGCGGATCCAGGTTG -3'		
5’- GAACCACCAGAACCATCGGG -3'	Confirmation of the integration of the complement vector into the genome	
5’- CAGTACGCGAAGAACCACGCC -3'		
5’- GGCGGACCTTTCCGGAGAGG -3'	*dut* probe amplification for Southern blot	
5’- CGGGGGCCAGTTCGACGTTC -3'		
5’- TTCCTGAGCACACCGTGACC -3'	SCO, DCO screening for *dcd:dut* mutant *M. smegmatis* strains	
5’- GTTCCGAGAATTGCTGCGACG -3'		
5'- CGCCAATTCACTTGCGTCTCAC -3'	A115F *dcd:dut* probe amplification for Southern blot	
5’- ACGTGACACCGATCGACTTGAGAT -3'		
5'- GCTGACGCACTCGACCTTCGGCTTCATCGATCCGGG -3'	QuikChange mutagenesis to create the A115F mutant Dcd:dut	
5'- CCCGGATCGATGAAGCCGAAGGTCGAGTGCGTCAGC -3'		
5’- TACTAAGATCTGCCCCGCCGGAAGGAGATATACATATGAGTAAAGGAG -3'	Cloning the C-terminal GFP fusion A115F *dcd:dut*	
5’- TAGTAAGATCTTCATTTGTATAGTTCATCCATGCC -3'		
5'- TCTCAACCGAAGAGTTCACC -3'	Primers for genomic uracil quantification	
5'- ATTCCGTAGTCATCCTGTGG -3'		
5'- GTACCTGGTGCGTCTGC -3'	Amplification of 1 kbp of *rpoB*	
5'- AGGCGGTAGGACTGACG -3'		
5'- TTTGTTTGTTTGTTTGTTTGGGCGGTGGAGGCGG -3'	Primers for dNTP pool measurements	
5'- TTATTATTATTATTATTAGGCGGTGGAGGCGG -3'		
5'- CCGCCTCCACCGCC -3'		
5’- CGTCGCCGATGGTCT G -3’	*sigA* cDNA amplification primers	
5’- CCACGCCCGAAGAGC -3’		
5’- CATACCGCACGGAATGGT -3’	*dut* cDNA amplification primers	
5’- TGATCAGCGAAACCTTGATCTC -3’		
5’- CTTCATCGATCCGGGCTTC -3’	*dcd:dut* cDNA amplification primers	
5’- CAGCTGCCCGATCTTCAT -3’		

5’- ATTAGGATCCAGTGCTGCTTTCCGATCGTGACATCC -3’	*M. smegmatis* Dcd:dut cloning primers
5'- ATTAAAGCTTTCAGGACTTGATGAAGTTGAGGTGCG -3'	
